# Tea and Tea-Drinking

**Published:** 1905-08-05

**Authors:** 


### Tea and Tea-Drinking.

Last year the Southwark Borough Council, at the request of Sir William Collins, gave permission for an inquiry to be made into the constituents of tea in order to ascertain what injurious ingredients were present, and if it were possible to obtain the benefits of tea without subjecting tea-drinkers to any of the deleterious symptoms. Accordingly Dr. Tebb, public analyst to the Borough of Southwark, was instructed to carry out the investigation. His report which has recently been published is worthy of attention, for not only does it emphasise the pernicious effects of excessive tea-drinking, but it conveys a startling impression of the magnitude of the evil as it exists at the present day. From statistics he computes that over six pounds of tea are consumed in this country each year per head of population. This means that the average person is in the habit of swallowing every day 3·6 grains of caffeine, and 9·7 grains of tannin—that is to say, half as much alkaloid and nearly as much tannin as the British Pharmacopœia regards permissible to be taken in occasional doses as a drug. When we add to these amounts the quantities of caffeine and tannin taken in the form of coffee and other beverages, it is possible to form a faint idea of the extent to which the present generation is saturating itself with these poisons. Members of the medical profession see often enough the effects in individual patients, who suffer from palpitations, dyspepsia, sleeplessness, emaciation, anæmia, and so forth; but it may be doubted if the damage done by tea is fully appreciated by any class in the community. Certainly no one would be more astonished than the pious old lady who seeking advice for indisposition was told that it was the result of intemperance. But, excessive tea-drinking can be so described, and it is probable that in the hypothetical case of the old lady, she would find it as difficult to abstain from

"The cups
That cheer but not inebriate,"

as the average British workman finds it to go without his beer.

The moral is that with the best possible intentions mankind will still err. Whether we believe or disbelieve that stimulants have played a beneficial part in the higher evolution of man, we must admit that men cannot, or, what is much the same thing in this twentieth century, will not resign the privilege of indulgence in them. This is the one starting-point whence philosopher and social reformer alike must proceed. Temperance in all things is, of course, easy to preach and not impossible to practice; but the lesson is one that can seldom be learnt except in the cradle and the nursery. Nevertheless, there is still a wide field for profitable endeavour. A field which has never yet been sufficiently appreciated, but which we commend to the activities of some future "Society for the Modification of Vice," whose objects shall be similar to those which were in Hogarth's mind when he drew his famous "Beer Street" and "Gin Lane," namely, so to regulate our vices as to rob them of their worst features.

For such a society there would be endless opportunities. The habitual drunkard is scarcely touched by the ordinary temperance reformer, but there is even some measure of salvation for the worst of all inebriates, the spirit drinker, if only he will transfer his affections to beer. Of what avail is it to upbraid the inveterate smoker? The real hope of improving him lies in supplying him with good tobacco which contains the least amount of volatile alkaloids and other noxious substances. A great part of the ill-results of excessive tea drinking may be attributed to the indigestion brought about by drinking the tea while it is too hot, and by allowing it to "stew" so that an undue proportion of tannin is extracted from the leaves. It might well be one of the aims of such a society as we have suggested to enlighten the public respecting the harm which can be done by the consumption of large quantities of alkaloids and tannin, and the means by which the excess of these substances can be diminished or avoided. It would be for the good of everyone if the fact were to be generally recognised that each kind of vice has its qualitative modifications, and that where it is not feasible to regulate the quantity of a certain excess, it is often possible so to regulate its nature as to rob the vice of the greater part of its sting.

